# Developing and Testing of Strain-Hardening Cement-Based Composites (SHCC) in the Context of 3D-Printing

**DOI:** 10.3390/ma11081375

**Published:** 2018-08-07

**Authors:** Hiroki Ogura, Venkatesh Naidu Nerella, Viktor Mechtcherine

**Affiliations:** 1 Institute of Technology, Shimizu Corporation, Tokyo 135-8530, Japan; ogura_h@shimz.co.jp; 2 Institute of Construction Materials, Technische Universität Dresden, 01602 Dresden, Germany; Venkatesh_naidu.nerella@tu-dresden.de

**Keywords:** digital concrete construction, 3D-concrete-printing, SHCC, ECC, fiber-reinforced concrete

## Abstract

Incorporating reinforcement into the practice of digital concrete construction, often called 3D-concrete-printing, is a prerequisite for wide-ranging, structural applications of this new technology. Strain-Hardening Cement-based Composites (SHCC) offer one possible solution to this challenge. In this work, printable SHCC were developed and tested. The composites could be extruded through a nozzle of a 3D-printer so that continuous filaments could be deposited, one upon the other, to build lab-scaled wall specimens without noticeable deformation of the bottom layers. The specimens extracted from the printed walls exhibited multiple fine cracks and pronounced strain-hardening characteristics under uniaxial tensile loading, even for fiber volume fractions as low as 1.0%. In fact, the strain-hardening characteristics of printed specimens were superior to those of mold-cast SHCC specimens.

## 1. Introduction

Digital concrete construction (DC) is usually carried out by means of an automated, selective, layer-by-layer deposition of fine-grained cementitious materials. Numerous research and commercial projects have been reported, including Contour Crafting [1], Concrete Printing [2,3] and CONPrint3D [4] and many others, all of which are often addressed using the generalized term 3D-concrete-printing. DC opens new horizons in construction, especially in terms of geometrical flexibility, reduction of costs and manpower as well as the dependence on it, increased productivity, speed of construction, construction in hazardous or remote areas, and sustainability [4,5]. The selective material deposition [1,2,3,4] by a printhead is seen at present as the DC approach most suitable for large-scale on-site applications. This approach is often referred to as extrusion-based, as opposed to the methods based on selective binding, see for example [6], even though in most known cases the concrete flow through a nozzle and its deformation after leaving the nozzle does not fully agree with the definition of conventional extrusion process [7]. Typically, the layer-by-layer deposition of fresh concrete occurs horizontally; thus, the structure grows vertically.

Most of the 3D-printing technologies developed as yet are focused on the placement of concrete, while the suggested solutions for incorporating reinforcement are still rudimentary. However, the understanding of the necessity of reinforcement for most structural applications is evident and various approaches have been suggested to deal with this issue. In the context of the selective material deposition techniques, extrusion-like methods, the following approaches have been proposed: (1) the placing of conventional steel reinforcement [8,9] or metal chains/spirals [1] horizontally between printed layers; (2) the incorporation of steel wire into the concrete filament in the printhead and deposition of the resulting composite [10,11]; (3) the envelopment of conventional [12,13] or 3D-printed [14] steel reinforcement in printable concrete; (4) the placing of conventional [9], post-tensioning [15,16] or customized steel reinforcement into cavities of the printed concrete elements, followed in many cases by filling of the cavities with flowable concrete; and (5) the mixing of short fiber into cementitious matrix before material deposition, thus using dispersed fiber reinforcement [17,18,19]. Each of these approaches has its advantages and disadvantages, and a discussion of this is not the purpose of this article, such that the authors refer to other recent publications, for example [14,20]. However, a general conclusion after examining the various techniques is that most of the proposed approaches (1) are discontinuous processes, meaning concrete printing and reinforcement placement must be carried out one after another; and (2) provide just a part of the reinforcement needed, for example, merely horizontal. Thus, different approaches must be combined to achieve the required degree of reinforcement.

The research at hand takes the approach of using dispersed, short-fiber reinforcement. This approach seems capable of being relatively easily integrated into the 3D-printing process by using fiber-reinforced concrete (FRC) instead of plain concrete. This straightforward approach is surely worth being pursued, especially taking into account the tremendous advances in the field of high-performance fiber-reinforced cement-based composites; see, e.g., [21,22]. Short fibers not only increase tensile and flexural strengths as well as the ductility of printed elements in the hardened state but also have the potential of mitigating plastic shrinkage and the related cracking. Since freshly 3D-printed elements are not protected by formwork—the deposited material is exposed to drying right from the moment of leaving the printhead and since the printable material is not supposed to exhibit any bleeding, indeed to delay plastic shrinkage, the mitigation of plastic shrinkage seems to be a critical issue here. Furthermore, while 3D-printing offers nearly unlimited geometrical freedom, the traditional reinforcement geometries, i.e., rebars and mesh reinforcement, hinder the use of the full potential of this new technology in terms of structural design and architectural forms. The integration of reinforcement directly into the deposited cement-based material seems to offer new options for functional, nature-imitating (bionic) structural design according to the principle of “form follows force”. In the context of digital fabrication, the FRC approach has been pursued by only a few researchers to date. Le et al. [2,23] used fine polypropylene fibers to exploit their potential in reducing shrinkage and deformation in the plastic state. The optimum mixture was concluded to be the one which passes through the extruder nozzle with the dosage of fibers of 0.13% by volume, as recommended by the supplier, and minimal binder content. Such fiber contents are typical in concrete technology when the purpose is the mitigation of plastic shrinkage.

Hambach et al. [17,18] used dispersed basalt fiber of 3 to 6 mm length and alternatively glass or carbon micro-fibers in cement paste and deposited the composite by means of a tiny nozzle. They reported a pronounced orientation of the fiber due to extrusion process and very high values of flexural strength, up to 100 MPa. Panda et al. [19] worked with various percentages of glass fibers of 3 mm to 8 mm length. Anisotropy and an increase in flexural strength were observed also in that study. Nematollahi et al. [24] investigated the effect of three types of fiber on the inter-layer bond and flexural strengths of 3D-printed geopolymer and reported increase in flexural strength when compared to reference geopolymer without fibers. These investigations confirm the feasibility of using dispersed fiber reinforcement as an approach to integrate reinforcement into the 3D-printing processes. The studies cited above focused on flexural strength only. While it is known that the flexural strength is positively affected by ductility, the ductility of 3D-printed FRCs as such was not examined. It should be underlined here that in many practical cases the ductility/deformability might be much more relevant than flexural strength.

With respect to structural performances, the use of strain-hardening cement-based composites (SHCC) is particularly promising [25]. Under uniaxial tensile loading SHCC exhibit quasi-ductile behavior with a strain capacity of up to several percent, resulting from the formation of multiple fine cracks prior to reaching the tensile strength of the composite. In addition to extremely high mechanical performance under quasi-static, cyclic [26] and dynamic [27] loadings, SHCC have a number of further advantages such as: (1) very narrow cracks typical of SHCC are favorable with respect to the durability of structural elements; (2) the relatively high content of fine polymeric fibers used in SHCC helps mitigate negative consequences not only of plastic shrinkage but also those of autogenous and drying shrinkage, thus preventing the formation of cracks, and in case cracks still form, controlling/preventing crack growth. To the best of the authors’ knowledge, very little research has been carried out to develop and test SHCC for use in 3D-printing processes. As of this writing, the only related publication was that by Soltan and Li [28], who optimized rheological behavior of SHCC containing 2% by volume of polyvinyl alcohol microfiber of 12 mm length and 40 μm diameter by applying certain binder compositions. The material was deposited manually using a caulk-gun apparatus with nozzle diameters of 8 to 13 mm. The uniaxial tensile tests showed that that the “printed” SHCC exhibited strain-hardening behavior superior to that measured for the cast specimens made of the same SHCC compositions.

In the framework of the research project presented in the article at hand, the authors have developed 3D-printable, strain-hardening, cement-based composites (PSHCC) by using various contents of high-density polyethylene (HDPE) microfibers. The choice of this fiber type instead of less expensive PVA fiber (as used in [28]) was made under consideration of superior mechanical properties of HDPE fiber which enable use of high-performance cement-based matrix without compromising strain-hardening performance of the composite (see, e.g., [29]). Such composites exhibit higher compressive strength, Young’s modulus and tensile strength and lower drying shrinkage, which are positive features, especially with respect to production of slender elements by 3D-printing. Furthermore, SHCC with HDPE fiber show finer crack patterns for the same fiber content, which is beneficial with respect to durability and serviceability. The adequacy of the mixtures is proven using 3D-printing of wall elements with a computer-controlled 3D-printing device. The fresh properties of SHCC were evaluated by means of flow table tests and ram-extrusion tests. With these testing techniques, an important step forward is made in comparison to the work by Soltan and Li [28] who used manual extrusion by means of a caulk-gun and simple flow table/drop-table test to characterize behavior of SHCC in a fresh state. Uniaxial tension tests were used to assess the mechanical performance of the hardened, printed SHCC. The results obtained were compared to those obtained for the selfsame SHCC compositions but produced using mold-casting. Moreover, investigation using a digital microscope was conducted to characterize the fiber distribution and orientation in the printed specimens.

It is noteworthy that at this stage 3D-printed SHCC was tested by applying tensile loading parallel to the longitudinal axis of the deposited filaments. While the proven strain hardening likely benefitted from preferential fiber orientation due to extrusion process, the authors are aware that the mechanical performance of PSHCC may be more modest for tensile loading acting lateral to the printed wall or even more so for the loading perpendicular to layer-to-layer interface. The authors intend to address the anisotropy of PSHCC elements in a follow-up paper, which should also present strategies to counteract pronounced anisotropy by material design and processing technique on one hand, and to wisely consider it in architectural and structural design on the other hand.

## 2. Experimental Program

### 2.1. Mixture Proportions and Raw Materials

Four SHCC mixtures were investigated, designated as Mixtures A to D; see Table 1. These mixtures had a water-to-binder ratio (W/B) of 0.22 to 0.24. The binder was composed of 75 wt % cement CEM II/A-M (S-LL) 52.5R, 15 wt % silica fume and 10 wt % fly ash. Silica fume was used to increase the viscosity, cohesion and thixotropy of the mixtures on one hand and to enhance the bonding between matrix and short fibers on the other. It was added as a slurry containing 50 wt % solid matter and 50 wt % water. High-density polyethylene microfibers were chosen for this study based on the positive experience of previous work on conventionally cast SHCC [21,22]. The length and diameter of the fiber were 6 mm and 0.012 mm, respectively. The HDPE fibers had a density of 0.97 g/cm${}^{3}$ and a tensile strength of 3000 MPa. Mixtures A and B contained 0.3% and 1.0% fibers, respectively, by volume of the composite, whereas Mixtures C and D contained 1.5% fiber.

As aggregate, a fine sand with a maximum particle size of 1.0 mm was used for all the mixtures. Such particle size is uncommonly large for SHCC; usually, the maximum aggregate size does not exceed 0.3 mm. The purpose of using larger particles in this investigation was that they should be helpful in achieving a higher yield stress of fresh mixture and consequently higher shape stability of the deposited material (see e.g., [30]). Additionally, the use of a larger maximum aggregate size enables the reduction of the required paste volume in the mixtures, in this fashion being helpful in reducing plastic shrinkage and drying shrinkage; the reduction being critical for formwork-free construction in 3D-printing. The quantity of sand had to be reduced with increasing fiber content, however, in order to provide for the sufficient extrudability of the mixtures. While Mixture A with 0.3 vol. % fiber had a sand-to-binder ratio (S/B) of 1.2; this proportion needed to be decreased to a mere 0.2 in Mixtures C and D, both of which had the highest fiber content of 1.5% by volume. All mixtures contained a polycarboxylate-based superplasticizer having a density of 1.06 g/cm${}^{3}$; 2.0% by mass of binder were added. The mixing was performed with a pan-type mixer having a capacity of 20 L. After dry materials (cement, sand, fly ash and fibers) were mixed at a speed of 25 rpm for 2 min, water and the silica fume slurry were added. After that, the materials were mixed with a velocity of 25 rpm for 2 min and subsequently at 45 rpm for 4 min.

### 2.2. Methods for Testing SHCC Properties in Fresh State

The rheological behavior of cementitious materials in the fresh state is crucial for DC applications. Rheological properties must be fine-tuned since they affect not only the pumping behavior (transport of the material to the printhead) [31,32], extrusion process [33,34], and the shape stability of the deposited filaments, but the quality of the interlayer bond and herewith the properties of final SHCC structure in a hardened state as well [18]. Le et al. [2] identified four key characteristics necessary for successful 3D-printing:

- Pumpability—The ease and reliability with which material is moved through the delivery system;
- Printability—The ease and reliability of depositing material through a deposition device;
- Buildability—The resistance of deposited fresh material to deformation under load;
- Open time—The period where the above properties are consistent within acceptable tolerances.

Later, Nerella et al. [35] reassessed these criteria and defined printability as a time-dependent compound property of pumpability, extrudability (defined as the ability of a material to be extruded through the nozzle with minimal energy consumption) and buildability.

There are few methods to evaluate the criteria named above. For example, Mechtcherine et al. [31] showed that the pumpability of fresh mixes can be assessed by so-called Sliper tests; Perrot et al. [36] described the test and evaluation methods for extrudability using a ram-extruder, while Perrot et al. [33] and Kazemian et al. [34] demonstrated that the buildability could be evaluated by applying compressive load to the material in fresh state. Wolfs et al. [37,38] and Suiker [39] presented an approach based on solid mechanics for predicting failure of 3D-printed concrete elements, combining experiments and numerical modelling techniques. Since a complete rheological characterization of printable SHCC is not an aim of this investigation, only the results of flow table testing and ram-extrusion testing are presented in this article, followed by the investigation of mechanical properties and SHCC microstructure.

Earlier researchers suggested that the existing flow tests on cement-based materials yield values which can be associated with their printability and buildability [34,40]. Thus, the flow values of each mixture were determined using a flow table (JIS R 5201:2015 [41], DIN EN 1015-3:2007-05 [42]), where initially a cone (dimensions 70 mm top diameter, 100 mm base diameter and 60 mm height) is filled with SHCC accompanied by slight tapping according to the standard. Then, the cone is lifted, allowing SHCC to spread on the plate while the plate remains steady. After the spread diameter is measured, the table is lifted-and-dropped 15 times in 15 s according to DIN EN 1015-3:2007-05 [42]. Flow value is the resulting increase in average post-shock spread diameter, given relative to the spread diameter before the shocks. The flow tests were conducted at an SHCC age corresponding to 20 min after the addition of water. The flow tests for few mixtures were repeated to check reproducibility. Spread after strokes differed by less than or equal to 3 mm only. Considering this, as well as the fact that flow tests are merely an empirical indicator of material yield stress, the flow tests were conducted only once for each remaining mixture, while extreme care in the testing procedure was taken. In this study, all tests on fresh SHCC, including 3D-printing, were performed 20 min after the time point of the addition of mixing water.

**Figure 1:**
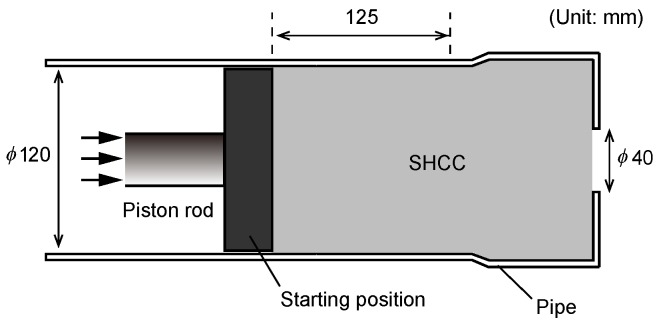
Schematic view of ram-extrusion test setup.

Extrudability is a critical aspect when it comes to applying fiber-reinforced concrete. Moreover, the addition of fibers pronouncedly modifies the rheological properties of fresh mixes. After a critical dosage limit is reached (this limit pronouncedly depends on the content of aggregates in SHCC mixtures in the first place, see also Table 1), fiber addition drastically reduces concrete flowability, whereas a similar influence can be expected in terms of extrudability. Furthermore, fibers may form dense networks and lead to blockages. The ram-extruder test may provides for the quantification of extrusion force, which could eventually be associated with both the extrudability and buildability of printable concretes. However, Nerella et al. [43] recently showed that the geometrical differences in ram-extruder (combination of plug flow and shear flow) and progressive cavity pump (shear flow at high shear rates) limit the significance of the ram-extruder tests. Notwithstanding this, the ram-extruder test is still a more appropriate means to assess extrudability than such purely empirical tests as slump flow test. Thus, in this study, extrudability was investigated using (a) ram-extrusion tests and (b) a 3D-concrete-printing test device (3DPTD), which enables both extrusion and buildability experiments. Figure 1 gives the schematic view of the custom-built ram extruder, consisting of a metal piston driven by a linear actuator. SHCC is filled into a cylinder of diameter 120 mm and height 300 mm and then extruded at a constant, pre-defined displacement rate through an orifice, a circular opening with a diameter of 40 mm. The total displacement of the piston during the experiments is 125 mm; see Figure 1 and corresponding force-displacement curves in Figure 7. A load-cell with maximum load capacity of 5 kN is mounted at the coupling of the piston and the linear actuator to measure the ram extrusion force. The ram-extruder tests were carried out 20 min after the addition of mixing water.

Specific steps in the ram-extrusion test procedure are as follows:

1. Filling SHCC into extruder cylinder in three steps, while
2. Consolidating by tapping 15 times using the tamping rod after each filling step;
3. Placing the piston into the pipe, and then
4. Placing the piston-cylinder assembly on the ram-extruder setup;
5. Connecting the piston rod to the loading device (linear actuator);
6. Extruding at a constant displacement rate of 15 mm/s by control with an electric motor (linear actuator).

**Figure 2:**
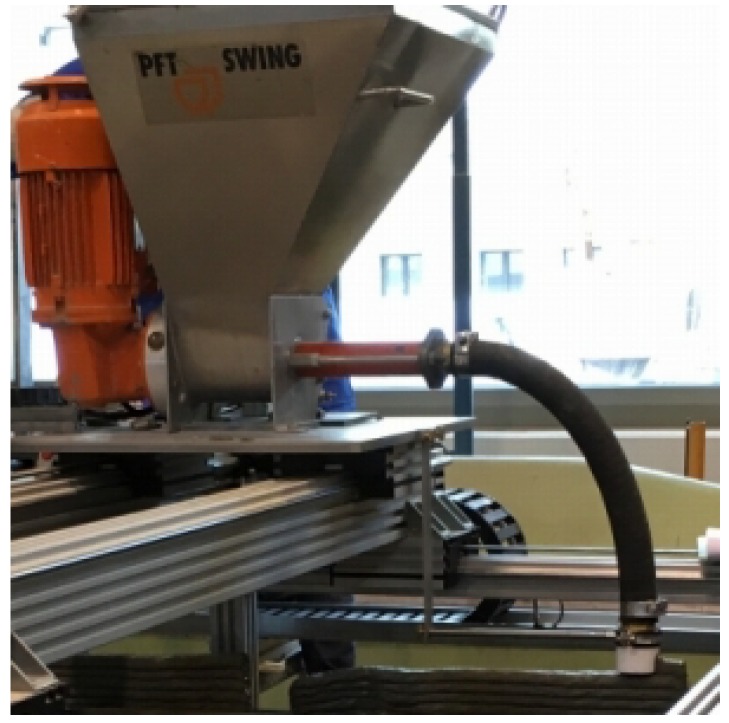
3D-concrete-printing test device (3DPTD).

The 3D-concrete-printing test device used in this study is shown in Figure 2. 3DPTD contains a computer-controlled, free-traversing printhead consisting of a container, a progressive cavity pump acting as extruder, a conveying pipe, and a nozzle for material deposition and forming. The screw pump is adopted to provide uniform pressure and can deliver materials up to maximum particle size of 2 mm. The nozzle has a cross-section of 18.72 mm by 30 mm. The machine can print concrete at different linear speeds and deposition rates. For purposes of this study, a linear printing speed of 50 mm/s was selected, based on preliminary extrudability investigations. Here, it must be noted that the extrudate flow rate (amount of concrete extruded per second) is synchronized with the velocity of the printhead. The time gap between depositions of two subsequent layers was set to one minute to reflect a critical case with respect to buildability, i.e., the case when the loading of 3D-printed layers increases with very short time intervals, similar to previous work [43]. The time gaps of one minute was the minimum possible interval for the given printing system; it includes time for printing the actual layer, moving the printhead back to its original position, visually inspecting material level in the hoper, condition of the printing setup, etc., before starting printing the next layer.

The buildability of the SHCC mixtures developed was verified with the help of direct printing tests using 3DPTD. SHCC walls of length 1000 mm, width 30 mm and height up to 120 mm were fabricated in one round. The wall height was the result of seven SHCC layers deposited one upon the other. The mixture tested was considered buildable if such a down-scaled wall retained its geometry and shape without observable deformations. Note that no accelerating agents were used in this study.

### 2.3. Manufacturing of Specimens

Mechanical properties were investigated on printed specimens made of the compositions Mixture B and Mixture C, both of which were proven extrudable and buildable; see Section 3.1. For comparison, additional SHCC specimens were cast in a conventional manner and tested as well.

**Figure 3:**
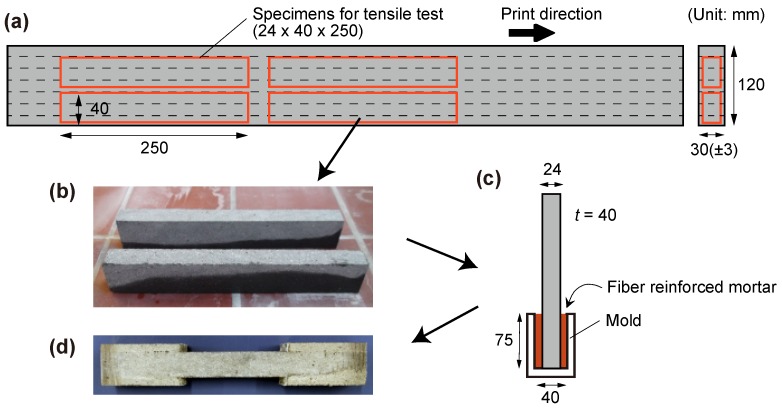
Preparation of specimens for tension tests: (**a**) position of the specimens in the printed wall; (**b**) cut specimens; (**c**) reinforcement for the specimens’ ends; and (**d**) specimens after reinforcement.

The seven layer-walls printed in the framework of buildability testing were eventually used for testing the mechanical properties of hardened SHCC. The minor unevenness and deformations (±3 mm) of the printed wall, resulting in some deviation from the rectangular form of cross-section, were therefore removed during the cutting process and have no influence on quantified strength values; see Section 3.2 and Section 3.3 The walls were printed at an SHCC-age corresponding to 20 min after the addition of mixing water. The extruded layers had nearly rectangular cross-sections provided by the shape of the nozzle. No compaction was applied in addition to that imposed by higher pressure during extrusion. After curing as described below, similar to previous studies [2,4], prism specimens with dimensions of 250 mm × 24 mm × 40 mm were saw-cut from the printed walls and used for uniaxial tension tests. The longitudinal axis of prism specimens was in the horizontal direction of the printed walls, hence parallel to interfaces between the layers; see Figure 3a. The ends of the specimens were strengthened by casting SHCC (W/B=0.20, 2% HDPE fiber), on both sides. In this way, dumbbell-geometry was produced to ensure that fracture localization occurred in the middle part of the specimen; see Figure 3c,d.

Both printable compositions, Mixtures B and C, were used as well to produce mold-cast specimens for the uniaxial tensile tests; see Section 2.4. The specimens were cast in dumbbell-shaped molds, each having an overall length of 250 mm and cross-sectional dimensions in the narrowed middle region of 24 mm by 40 mm. In addition, cubic specimens with an edge length of 100 mm were cast for the compression tests. No vibration for compacting was applied. However, after filling half the height of the mold with SHCC, slight consolidation/distribution of filled SHCC occurred by means of a tapping rod (15 times). Then, the mold was filled completely, and the consolidation step was repeated.

All specimens (both mold-cast and printed) were cured in water for seven days starting with SHCC age of 24 h. After that, they were stored in a climatic chamber at a constant temperature of 20 °C and a relative humidity of 65%. At an age of 22 days, the samples were cut from the printed walls using wet-grinding and subsequently strengthened by casting SHCC at both the ends. After the casting, printed specimens were stored again in the climatic chamber. Both the printed and casted specimens were under the self-same curing conditions up to 22 days. Since CEM II/A-M (S-LL) 52.5 R cement was used, most of the hydration should have already completed at this age. The slight variation of surrounding conditions after 22 days is unavoidable, as the printed specimens “must” be cut and prepared for the mechanical testing. All specimens were removed from the climatic chamber at an age of 26 days. Both tension and compression tests were conducted at an age of 28 days.

### 2.4. Method for Uniaxial Tension Tests

**Figure 4:**
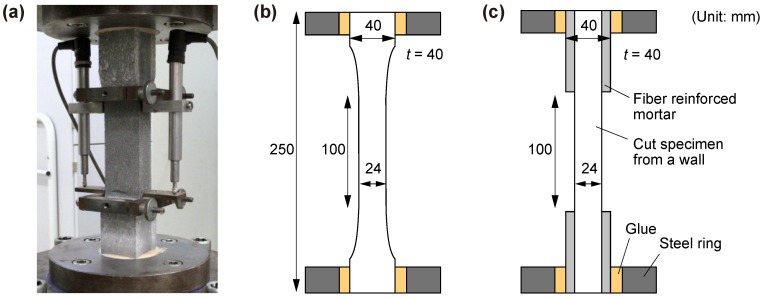
(**a**) setup for uniaxial tension tests; (**b**) schematic view of uniaxial tension tests on mold-cast specimen and (**c**) on printed specimen.

The uniaxial tension tests were performed in a manner similar to those presented in earlier works at the TU Dresden [22] in an Instron machine (Darmstadt, Hessen, Germany) with a load capacity of 100 kN in a deformation-controlled mode with a displacement rate of 0.05 mm/s. Non-rotatable boundary conditions were ensured by gluing the samples at both ends in 20 mm thick steel rings, which were then bolted to the testing machine. The deformations were measured on a 100 mm gauge length in the middle of the specimens with two Linear Variable Differential Transformers (LVDTs) fixed on a built frame, as shown in Figure 4a.

As detailed in previous sections, mold-cast, dumbbell-shaped samples of cross-sectional dimensions of 24 mm by 40 mm were used as reference (see Figure 4b). The printed specimens were saw-cut prisms with eventually strengthened ends (see Figure 4c).

## 3. Results and Discussion

### 3.1. Properties of SHCC in Fresh State

The results of the flow table tests are summarized in Table 2. Though all the mixtures have similar spreads before strokes $F_{0}$, the spread after 15 shocks $F_{1}$ varies. The difference is clearly readable in the relative spread $F_{r}$, which was calculated by considering flow values after and before shocks.

**Figure 5:**
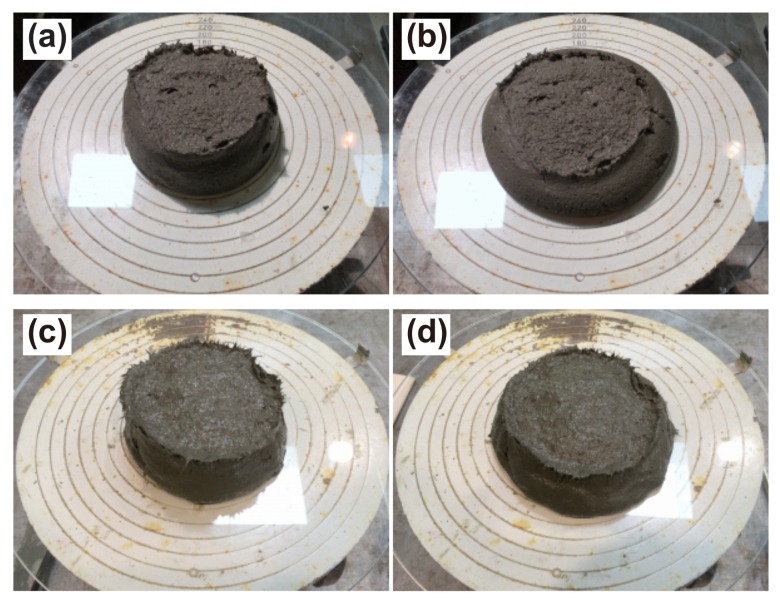
Selected results of the flow table tests (**a**) before and (**b**) after shocks for Mixture A as well as (**c**) before and (**d**) after shocks for Mixture C.

After shocks both Mixtures A and Mixture D have higher flow spread diameters and higher $F_{r}$ values in comparison to Mixtures B and C, which may be traced back to the higher W/B of Mixtures A and D. Figure 5 shows exemplarily the spread test images for Mixtures A and C. While Mixture A has a considerably lower fiber content, just 0.3% by volume in comparison to Mixture C at 1.5%, higher sand content, lower content of binder and higher W/B, the flow values before shocks were equal for these two mixtures. This was not so after strokes, as the spread of Mixture A after the shocks was larger than that of Mixture C. It seems that the higher W/B is the deciding factor in that difference. The general trend is that, with an increasing fiber content, ever more sand had to be replaced by binder and water to keep the workability at the same level. Mixture D has the higher spread diameter despite high fiber content, which was achieved at the lowest S/B and highest W/B. In fact, visual observation during production handling of mixtures confirmed the flow spread measurements: a decrease in viscosity and cohesion of mixture D was very evident when compared to Mixture C, i.e., the mixture with the same fiber content of 1.5% and the same S/B=0.2, but lower W/B. A previous parametric study by the second and third authors on a vast range of plain concretes indicated that mixtures with spread values above 140 mm after strokes are not buildable [44]. Based on that observation and the flow spread results of the study at hand, Mixtures B and C can be expected to be more suitable for the 3D-printing in terms of buildability.

**Figure 6:**
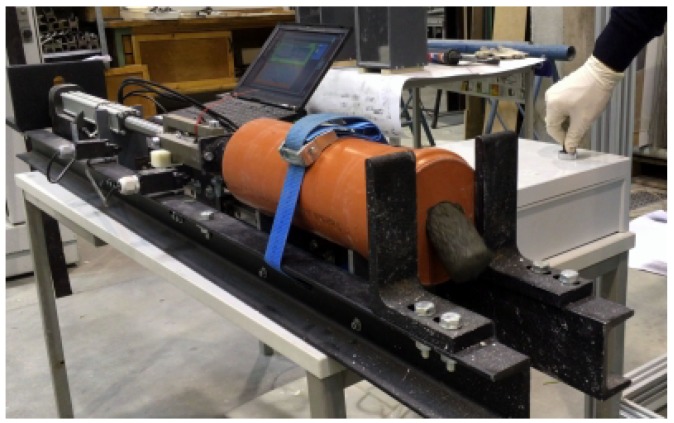
Ram-extrusion device in operation, here Mixture B is extruded.

To estimate risk of blockages, a ram-extruder device was used. Figure 6 shows this device in operation at the displacement rate of 15 mm/s applied in this investigation. All SHCCs could be extruded through orifice-building-consistent filaments. No blocking of the flow occurred even for the relatively stiff compositions, Mixture B and Mixture C. Higher fiber content as well proved not to cause blocking during ram-extrusion.

**Figure 7:**
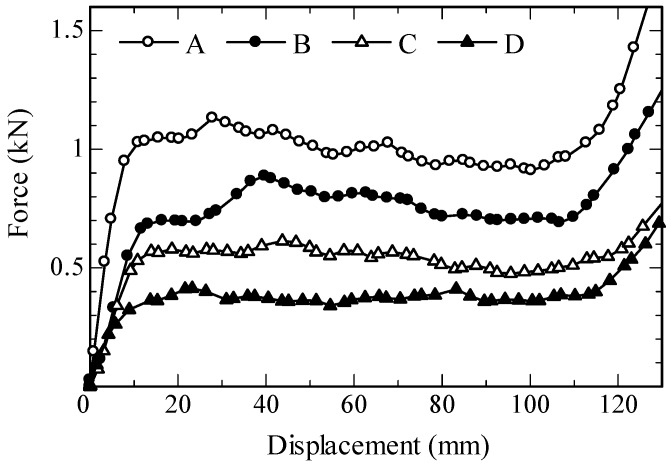
Force vs. piston displacement curves obtained in ram-extrusion tests.

Figure 7 shows the extrusion force–piston displacement curves obtained from the ram-extrusion tests. The easily distinguishable characteristic of these curves is that of their three distinct segments, corresponding to three different stages in the experiment. Note that the observed behavior is similar to the results reported in earlier works on ram extrusion [36,45]. According to Perrot et al. [36], the three parts can be attributed to (a) Part I: compaction of the tested material at flow initiation; (b) Part II: frictional resistance during equilibrium plug flow and (c) Part III: compaction against static/unshared concrete, the so-called “dead-zone”. Thus, for the first and third part of the curve, the load exponentially increases with increasing displacement of the piston, while, in the second part, starting at a displacement of approximately 10 mm, the force remains almost constant or even decreases slightly as displacement increases. This slight decrease in force is an expected result since the total frictional resistance decreases with decreasing billet length [36,45]. For the comparative assessment, only Part II is relevant [35]. The results show that the maximum extrusion force changes depending on the extruded material in the range between 0.4 and 1.1 kN.

Interestingly, the extrusion force appears to decrease with increasing fiber content. This observation directly contradicts the common understanding that increasing fiber dosage affects the flowability of FRC negatively, and thus potentially decreases extrudability as well. On closer examination, however, it is evident that the extrusion force, measured with the ram-extruder results presented in this paper, is governed more by the sand-to-binder ratio than by the fiber content. The S/B of Mixtures A, Mixture B and Mixture C were 1.20, 0.50 and 0.20, respectively; see Table 1. Higher relative sand content at lower relative binder contents means more inter-particle friction, which subsequently leads to higher plastic viscosity of concrete and higher frictional resistance at the concrete-extruder wall interface. At the same time, higher sand content also increases the probability of the sand particles’ interlocking at the piston/wall interfaces [35]. In fact, a rubbing noise from the interface during extrusion of Mixture A was loudest. As a consequence Mixture C with the lower S/B ratio showed lower extrusion load in comparison with Mixture A and Mixture B. Increasing W/B at a constant S/B ratio as well as constant fiber and superplasticizer content leads to further decreases in the extrusion force, as observed for Mixture D in comparison to Mixture C; see Figure 7.

The results of ram-extruder tests suggest that all tested mixtures may be suitable for the 3D-printing from the extrudability perspective. However, this conclusion does not consider differences in extrusion devices, i.e., ram-extruder vs. screw-extruder. The results obtained by means of screw-extruder as employed in the 3DPTD are presented in the following subsection.

### 3.2. Printing Tests

**Figure 8:**
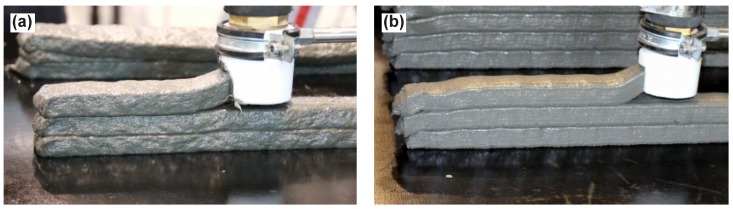
3D-concrete-printing device placing (**a**) Mixture B and, for sake of comparison, (**b**) lightweight fine-grained concrete.

**Figure 9:**
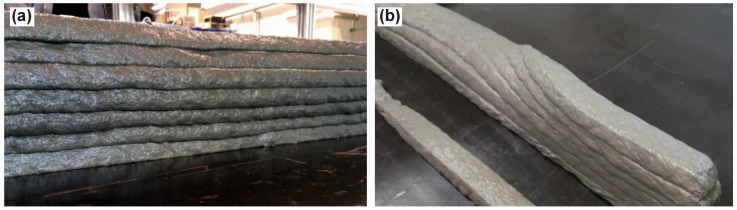
Seven-layer printed specimen with interlayer time interval of one minute: (**a**) Mixture B; and (**b**) Mixture D.

3D-printing tests were conducted using Mixtures B and C only, since these two compositions seemed to be most promising as based on the interpretation of flow table test results with respect to buildability; see Section 3.1. Actually, Mixture D was tried as well, but buildability in 3DPTD experiments was not sufficient; see also Figure 9b. Mixture A was not further optimized since its content was patently insufficient with respect to the mechanical performance expected of SHCC. Figure 8a shows the 3D-concrete-printing device in operation while depositing Mixture B; a 3D-printed, fine-grained lightweight aggregate concrete without fibers is shown in Figure 8b for comparison. This material was investigated in a parallel study and will not be considered here further. The comparison shows, however, that the addition of a high percentage of fiber obviously makes it more difficult to obtain a smooth surface on the deposited filaments.

Figure 9a demonstrates seven-layer printed specimens produced using Mixture B; Mixture C showed similar buildability performance and so is not shown here. Both mixtures could be printed through a nozzle with consistent filaments to build up to seven layers in one session without noticeable deformation of bottom layers. Both mixtures could be printed through a nozzle with consistent filaments. Printed layers were visually observed (qualitative, with a precision of ±3 mm based on the nozzle-layer separation distance) for consistency and buildability. If the SHCC was not buildable, then the printed layer deformed either due to its self-weight (spreading) or due to the vertical load acting upon from the upper layers; see Figure 9b. This was not the case for Mixtures B and C. In addition to visual examinations, at the age of 24 h, the heights of the printed walls were measured to quantify the final deformations. The final heights of 7-layer walls printed with Mixtures B and C were 120 mm, which means a 11.04 mm discrepancy between the expected height (nozzle opening of 18.72 mm multiplied by the number of layers) and the measured height of the walls. Such discrepancy of a 1.58 mm/layer or approximately 8.2% was likely due to incomplete filling of the nozzle or plastic deformations in the process of filament deposition. Since the wall thickness was consistent for all printed layers, i.e., the top and the bottom layers had same thickness, the deformations after the material deposition due to vertical loads induced by upper layers can be excluded. In the authors’ opinion, such discrepancies or deformations are non-critical with respect to possible collapse/buckling of the printed elements or inconsistent wall thickness. However, the knowledge of the exact height of filaments is absolutely essential for purposeful planning and the steering of the 3D-printing process. In contrast to Mixtures B and C, Mixtures A and D were characterized “not buildable” since the walls exhibited pronounced deformations while being printed and eventually collapsed. Further investigations are needed to isolate the exact origins of the discrepancies and to further improve the buildability of developed PSHCCs. The printed layers were free of surface defects, including any discontinuity due to extrudability deficiencies and/or inadequate cohesion. The layer edges were nearly rectangular. Although there was concern about the possibility of cracking due to plastic shrinkage, neither at an early age of 24 h nor at a later age of 24 days were any cracks were observed on the surface of printed specimens. Thus, it was proven that Mixture B and Mixture C as developed accord with expectations concerning the printability of SHCC. These mixtures were further investigated with respect to their mechanical properties.

As indicated above and as estimated based on flow table test results, i.e., flow spread of 153 mm after strokes and [44], Mixture D could not be printed in a desired regime, i.e., with short time intervals of one minute only between depositions of two subsequent layers. A collapse occurred after printing six layers; see Figure 9b. The collapse appears to be caused by deviation of the center of gravity from the longitudinal symmetry axis of the wall element due to deformation of the lowest layer under the action of vertical loads from upper layers. The deformation was clearly visible, which confirms that the static yield stress of Mixture D at an age of 20 min was not sufficient for 3D-printing in the chosen regime.

### 3.3. Mechanical Properties

Values of first-crack stress, tensile strength and strain capacity as obtained from uniaxial tension tests are given in Table 3 along with compressive strength values measured in the compression tests. The first-crack stress is defined as the stress at which the stress–strain response in uniaxial tension test clearly deviates from linearity as a result of crack formation. Higher fiber content in Mixture C is probably the reason for slightly higher first-crack stress and tensile strength of this composition in comparison to Mixture B, especially in the case of cast specimens. The positive effect of higher fiber content on first-crack stress can be traced back to the fact that more numerous micro-fibers can better prevent growth of micro-cracks in SHCC in comparison to the case with less numerous micro-fibers. This holds true also for the control of larger cracks which form after stress level further increases and which cross the entire specimen. Higher number of fibers crossing a crack results in higher bridging forces, thus increasing ultimate stress magnitude needed to overcome this bridging action. Additionally, one can see that the scatters both in the first-crack stress and tensile strength are considerably higher for the cast specimens. Compressive strength was measured on cast specimens only. With 105 MPa for Mixture B and 104 MPa for Mixture C, the values were practically equal.

**Figure 10:**
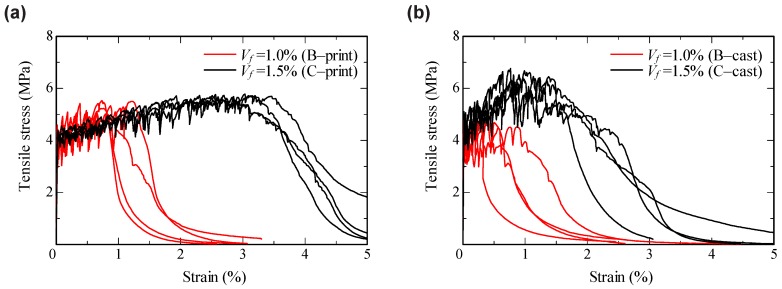
Stress–strain curves obtained from uniaxial tension tests on (**a**) printed specimens; and (**b**) mold-cast specimens.

**Figure 11:**
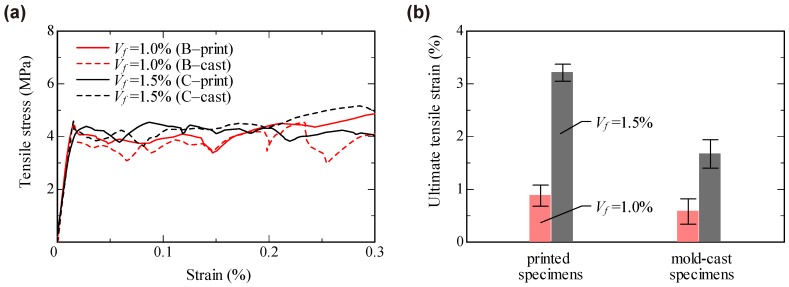
Results of uniaxial tension tests: (**a**) stress–strain curves up to strain level of 0.3%; and (**b**) ultimate tensile strain (strain capacity).

Figure 10 shows the stress–strain relationships obtained from the uniaxial tension tests. The strain was calculated from displacements measured with a 100 mm gauge length LVDTs in the middle of the specimens. All specimens showed strain-hardening behavior accompanied by the formation of multiple, closely spaced fine cracks. One can see that the data scatter is more pronounced for the results obtained for the mold-cast specimens. Figure 11a shows representative stress–strain curves for all parameter combinations up to a strain level of 0.3% to compare the material behavior before and at the beginning of crack formation. Obviously, the manufacturing method had no pronounced influence on either the elastic properties of the composites or their performance immediately after formation of the first cracks.

The difference in behavior was much more pronounced at higher strain levels. Table 3 and Figure 11b show the ultimate tensile strain, indeed strain capacity, which is the strain at reaching tensile strength (stress maximum) and, thus, the strain just before the onset of the descending branch. The ultimate tensile strain was found to increase according to the fiber volume fraction. This can be explained by increase in total crack-bridging force with increasing number of fibers intersecting each crack. For cast specimens, the ultimate strain for SHCC with a fiber content $V_{f}$ of 1.5% was about three times higher than that measured for SHCC with $V_{f}=1.0\%$. In the case of printed specimens, the difference was even more pronounced, nearly four times. Furthermore, the printed specimens exhibited higher values of the ultimate strain and smaller scatter of the results in comparison to those of the mold-cast specimens. Note that the scatter of the results obtained in this study is significantly lower than that in the previous research [28].

**Figure 12:**
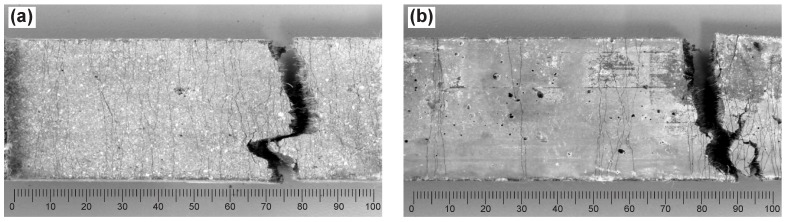
Representative crack patterns of specimens after failure in uniaxial tension tests: (**a**) C-print specimen, and (**b**) C-cast specimen.

Figure 12 shows representative surface crack patterns observed on the specimens made of Mixture C ($V_{f}=1.5\%$). While the printed specimens (C-print) exhibited well-distributed, fine cracks, the crack pattern of the mold-cast specimens (C-cast) was less regular, less “saturated”. Both materials exhibited almost the same crack widths of approximately 50 μm on average.

**Figure 13:**
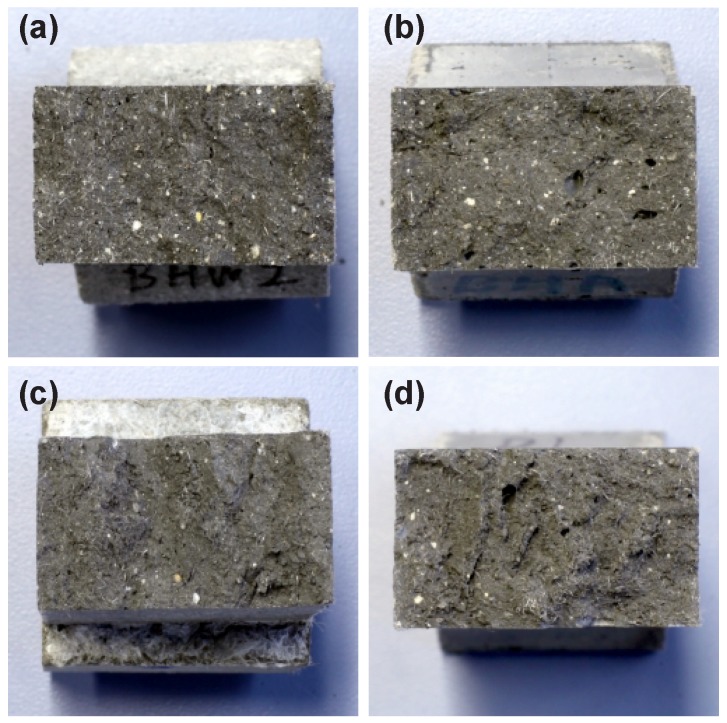
Fracture surfaces after failure of specimens in uniaxial tension tests: (**a**) B-print; (**b**) B-cast; (**c**) C-print; and (**d**) C-cast.

Additionally, the fracture surfaces of the specimens were examined. The fiber distribution was found to be uniform in all specimens. However, mold-cast specimens (B-cast and C-cast) had more relatively large voids when compared to the printed specimens, B- and C-print; see Figure 13. The lower porosity of the extruded composites, when compared to mold-cast specimens, has also been noted in previous studies; see e.g., [23]. This seems to be due to the mechanical compaction during the extrusion process. The presence of larger voids in cast specimens may explain the higher scatter of the first-crack stress and the ultimate tensile strain values observed for these samples. Furthermore, they indicate a possible reason for lower ultimate strain values in comparison to the printed specimens: large voids lead to early localization of failure, which occurs before a saturated crack pattern could develop.

**Figure 14:**
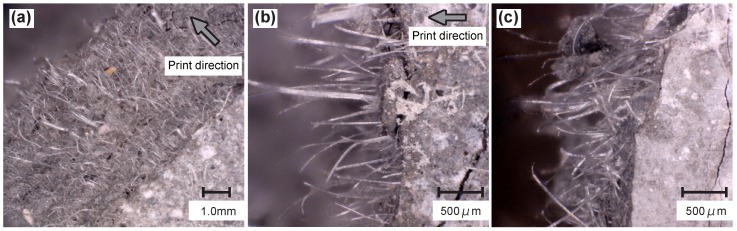
Microscopic images of fracture surfaces of: (**a**,**b**) C-print specimens; and (**c**) C-cast specimen.

Another object of examination on fracture surfaces was fiber orientation. Figure 14a,b presents images of the fibers on the fracture surfaces of the printed specimens captured by means of digital microscope VHX-6000 produced by KEYENCE (Osaka, Japan). Fiber distribution appears uniform and it seems that most fibers are oriented in a direction perpendicular to the crack surface, i.e., in the direction of extrusion. In contrast, fibers observed on the fracture surface of the mold-cast specimens appear more randomly oriented; see Figure 14c. These findings are consistent with results from previous studies on 3D-printing using fiber-reinforced, cementitious composites [17,46]. Thus, fiber orientation was likely influenced by the printing process. Kunieda et al. [47] suggested that, based on numerical analysis in which short fibers were discretized, fiber orientation is associated with ultimate tensile strain. According to said analysis, the fiber orientation aligned with the direction of tensile loading as observed in the tests on printed specimens must lead to an increase in ultimate tensile strain in comparison to mold-cast specimens exhibiting a much more random fiber orientation.

## 4. Conclusions

Printable, Strain-Hardening, Cement-based Composites (PSHCC) with HDPE fiber contents of 1% and 1.5% suitable for digital construction were developed and characterized. The results of the research at hand can be summarized as follows:

(1) The PSHCC mixtures developed could be printed by depositing consistent filaments with a time interval of one minute between subsequent layers; wall elements with a height of 120 mm could be built without any noticeable deformation of the bottom layers.
(2) The shape stability of the printed wall elements could be roughly estimated from the results of flow table tests.
(3) Extrudability, as quantified by means of a ram extruder, depends on the sand content of the mixture in the first place. The ease of extrusion increases with decreasing sand content.
(4) The specimens extracted from the printed walls exhibited pronounced strain-hardening behavior under uniaxial tensile loading for fiber concentrations as low as 1%. For a fiber content of 1.5%, strain capacity was considerably higher, and very uniformly distributed fine multiple cracks were observed.
(5) Printed specimens exhibited superior strain-hardening behavior and more pronounced multiple cracking in comparison to mold-cast specimens made of the selfsame SHCC mixtures. This can be likely traced back to (1) the absence of large air voids in printed specimens due to compaction in the printhead; and (2) the favorable orientation of fibers as a result of the extrusion process.

The ongoing work of the researchers is to focus on (1) detailed characterization of the time-dependent rheological properties of PSHCC in the fresh state and their relation to the process parameters of 3D-printing; (2) quantifying mechanical properties of layer-to-layer interfaces and their effect on the anisotropy of printed SHCC; and (3) comprehensive investigation of the microstructure of printed SHCC.

## Figures and Tables

**Table 1:** Mixture proportions for composites under investigation.

Mixture Name	W/B	S/B	Fiber Volume Fraction (vol.%)
A	0.24	1.20	0.3
B	0.22	0.50	1.0
C	0.22	0.20	1.5
D	0.24	0.20	1.5

**Table 2:** Results of flow table tests at 20 min after mixing.

Mixture	Flow Spread Diameter		Relative Spread $F_{r}\;=\;\left(F_{1}/F_{0}\;-\;1\right)$
	Before Shocks $F_{0}$ [mm]	After Shocks $F_{1}$ [mm]	
A	119	142	0.19
B	120	135	0.13
C	119	133	0.12
D	129	153	0.19

**Table 3:** Results of the mechanical tests—mean values: coefficients of variation are given in parentheses.

	First-Crack Stress (MPa)	Tensile Strength (MPa)	Ultimate Tensile Strain (%)	Compressive Strength (MPa)
B-print	4.18 (0.10)	5.32 (0.04)	0.88 (0.23)	—
B-cast	4.09 (0.15)	4.53 (0.10)	0.58 (0.41)	105 (0.12)
C-print	4.25 (0.03)	5.66 (0.02)	3.21 (0.05)	—
C-cast	4.63 (0.09)	6.32 (0.04)	1.67 (0.16)	104 (0.02)
